# FAMILY CAREGIVERS’ PSYCHOLOGICAL WELL-BEING AND PHYSICAL HEALTH IN THE CONTEXT OF THE WORK ENVIRONMENT

**DOI:** 10.1093/geroni/igac059.2549

**Published:** 2022-12-20

**Authors:** Barbara Hodgdon, Jen Wong

**Affiliations:** The Ohio State University, Columbus, Ohio, United States; The Ohio State University, Columbus, Ohio, United States

## Abstract

As working family caregivers navigate work and care responsibilities, the work environment may contribute to their psychological well-being and physical health outcomes. Working caregivers who provide multi-generational care (e.g., sandwiched caregivers) experience greater vulnerability when compared to non-sandwiched caregivers (e.g., filial caregivers). This vulnerability could be mitigated by supportive work characteristics (e.g., greater decision authority and supervisor support). Informed by the life course perspective, this study compared the psychological well-being and physical health of working sandwiched and filial caregivers, and the moderating role of work characteristics (decision authority and supervisor support). Sandwiched (n=80; Mage=46.3) and filial (n=62; Mage=55.7) caregivers from the Midlife in the United States (MIDUS-II) Survey provided information about their background, caregiving, employment, well-being, and health via a set of questionnaires and phone interview. Regression analyses showed that sandwiched caregivers exhibited lower levels of generativity than filial caregivers. Moderation analyses revealed that sandwiched caregivers with greater decision authority trended toward exhibiting greater autonomy than other caregivers. Sandwiched caregivers with greater decision authority also exhibited significantly less difficulty with instrumental activities of daily living (IADLs) than other caregivers. Finally, sandwiched caregivers with greater than average supervisor support trended toward exhibiting higher level of generativity as compared to other caregivers. Results highlight the impact of sandwiched caregiving on autonomy, generativity, and IADLs in the context of decision authority and supervisor support. Findings may inform workplace programs and policies aimed at enhancing caregivers’ sense of decision making and increasing supervisor support in order to promote caregivers’ psychological well-being and physical health.

